# NOXA-dependent contextual synthetic lethality of BCL-XL inhibition and “osmotic reprogramming” in colorectal cancer

**DOI:** 10.1038/s41419-020-2446-8

**Published:** 2020-04-20

**Authors:** Gertrud Knoll, Petra Riffelsberger, Danielle Raats, Onno Kranenburg, Martin Ehrenschwender

**Affiliations:** 1 Institute of Clinical Microbiology and Hygiene, University Hospital Regensburg, Franz-Josef-Strauss-Allee 11, 93053 Regensburg, Germany; 2 Department of Surgical Oncology, UMC Utrecht Cancer Centre, PO Box 85500, 3506 GA Utrecht, The Netherlands

**Keywords:** Cancer microenvironment, Cancer therapy, Apoptosis

## Abstract

A sophisticated network of BCL-2 family proteins regulates the mitochondria-associated (intrinsic) apoptosis pathway. Antiapoptotic members such as BCL-XL or MCL-1 safeguard the outer mitochondrial membrane and prevent accidental cell death in a functionally redundant and/or compensatory manner. However, BCL-XL/MCL-1-mediated “dual apoptosis protection” also impairs response of cancer cells to chemotherapy. Here, we show that hyperosmotic stress in the tumor environment abrogates dual BCL-XL/MCL-1 protection. Hypertonicity triggers upregulation of NOXA and loss of MCL-1 and thereby enforces exclusive BCL-XL addiction. Concomitant targeting of BCL-XL is sufficient to unlock the intrinsic apoptosis pathway in colorectal cancer cells. Functionally, “osmotic reprogramming” of the tumor environment grants contextual synthetic lethality to BCL-XL inhibitors in dually BCL-XL/MCL-1-protected cells. Generation of contextual synthetic lethality through modulation of the tumor environment could perspectively boost efficacy of anticancer drugs.

## Introduction

Elimination of cancer cells mainly relies on activation of the mitochondria-associated (intrinsic) apoptosis pathway. This cell death modality is tightly controlled by a complex network of BCL-2 family proteins. The effector molecules BAX and/or BAK trigger mitochondrial outer membrane permeabilization (MOMP), release of cytochrome c, caspase activation and ultimately condemn a cell to death. BAX and BAK are kept in check by BCL-2, BCL-XL and/or MCL-1 (collectively also referred to as BCL-2-like proteins)^1^. Targeting BCL-2-like proteins emerged as a therapeutic strategy and spurred development of “BH3 mimetics”^2^. This novel class of anticancer compounds mimics the function of BH3-only proteins (the natural antagonists of BCL-2-like proteins) and unlocks the intrinsic apoptosis pathway. The selective BCL-2 inhibitor ABT-199 (venetoclax) proved successful in clinical trials and is meanwhile approved for the treatment of certain leukemia^3^. However, functional redundancy and compensatory roles among the antiapoptotic BCL-2-like proteins can establish “codependencies” (e.g. BCL-XL/MCL-1) that hamper efficient cancer cell elimination when a single BCL-2-like protein is targeted. Preclinical studies demonstrated that inhibition of two or more BCL-2-like proteins was required to overcome this limitation^4–7^. Our previous work showed that cancer cells facing a hypertonic environment exhibited a lower threshold for MOMP induction^8–10^. We hypothesized that this could reflect hypertonicity-induced alterations in the BCL-2 family network. How hyperosmotic stress affects cancer cells has not been comprehensively investigated, but reports document enhanced cisplatin sensitivity, secretion of angiogenesis-promoting cytokines and upregulation of resistance- or metastasis-associated proteins^11–14^.

Here, we show that hypertonicity can alter codependencies of cancer cells on BCL-2-like proteins. Hyperosmotic stress shrinks dual BCL-XL/MCL-1 protection to exclusive BCL-XL addiction by upregulation of NOXA and concomitant loss of MCL-1. This renders selective BCL-XL inhibition sufficient for activation of the intrinsic apoptosis pathway. Functionally, “osmotic reprogramming” of the tumor environment grants contextual synthetic lethality to BCL-XL inhibitors in dually BCL-XL/MCL-1-protected cancer cells. Perspectively, our findings could boost the efficacy of anticancer drugs.

## Results

### BCL-XL/MCL-1 coinhibition triggers BAX/BAK-dependent apoptosis in colorectal cancer cells

**Fig. 1:**
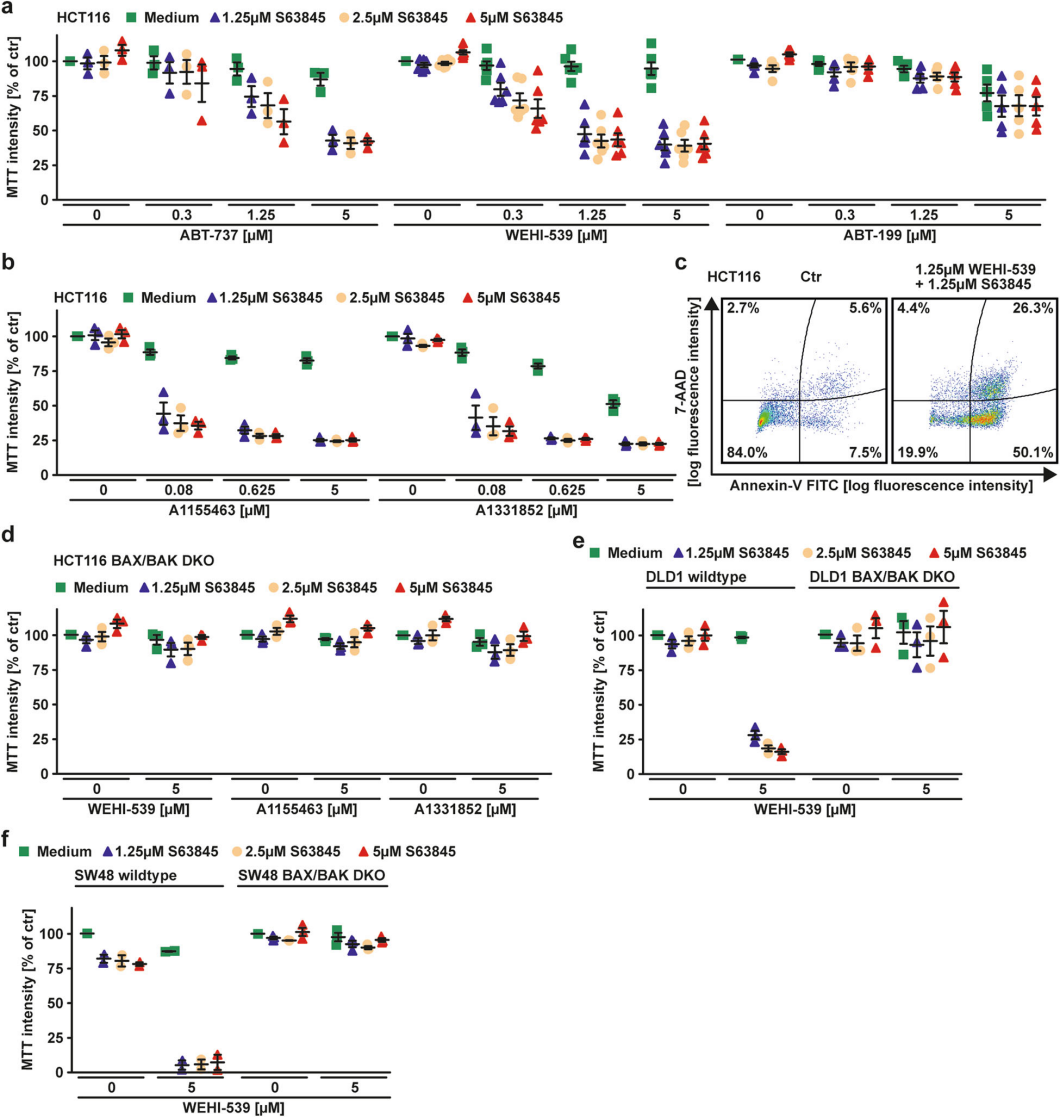
BCL-XL/MCL-1 coinhibition triggers apoptosis in colorectal cancer cells. **a**, **b** Cells were challenged with the indicated concentrations of **a** ABT-737, WEHI-539 and ABT-199 or **b** A1155463 and A1331852 for 18 h in the presence and absence of the MCL-1 inhibitor S63845. **c** HCT116 cells were challenged with WEHI-539 and S63845 for 6 h and subsequently analyzed by flow cytometry for 7-AAD- and annexin-V positivity. **d**–**f** BAX/BAK-deficient HCT116 cells, DLD1 cells, SW48 cells and BAX/BAK-deficient variants thereof were challenged with the indicated concentrations of WEHI-539, A1155463 and A1331852 for 18 h in the presence and absence of the MCL-1 inhibitor S63845. For (**a**, **b**, **d**–**f**), data points and mean ± SEM from three independent experiments are shown; for (**c**), data shown are representative of two experiments performed.

Challenging HCT116 cells with ABT-737 (targeting BCL-2, BCL-XL and BCL-W), WEHI-539 (targeting BCL-XL) and ABT-199 (targeting BCL-2) only marginally killed cancer cells (Fig. 1a)^15–18^. However, combination with the MCL-1-selective inhibitor S63845^18,19^ drastically enhanced ABT-737 and WEHI-539 (but not ABT-199) cytotoxicity (Fig. 1a, e, f) and even synergized in cell death induction (Supplementary Table 1). This suggested a BCL-XL/MCL-1 codependency and implicated that both (a) were involved in maintaining integrity of the outer mitochondrial membrane and (b) exhibited functional redundancy with compensatory roles. Expectedly, combinatorial treatment with S63845 and other BCL-XL-selective inhibitors (A1155463 or A1331852) also synergistically induced cell death (Fig. 1b, Supplementary Table 1)^20,21^. Coinhibition of BCL-XL/MCL-1 resulted in annexin-V/7-AAD positivity (Fig. 1c) and critically depended on the pore-forming proteins BAX and BAK for cytotoxic effects (Fig. 1d–f). In sum, our results indicated that BCL-XL and MCL-1 safeguarded mitochondrial integrity in a functionally redundant manner, which necessitated dual BCL-XL/MCL-1 inhibition for efficient activation of the intrinsic apoptosis pathway.

### Hyperosmotic stress renders selective BCL-XL inhibition sufficient for cell death induction

**Fig. 2:**
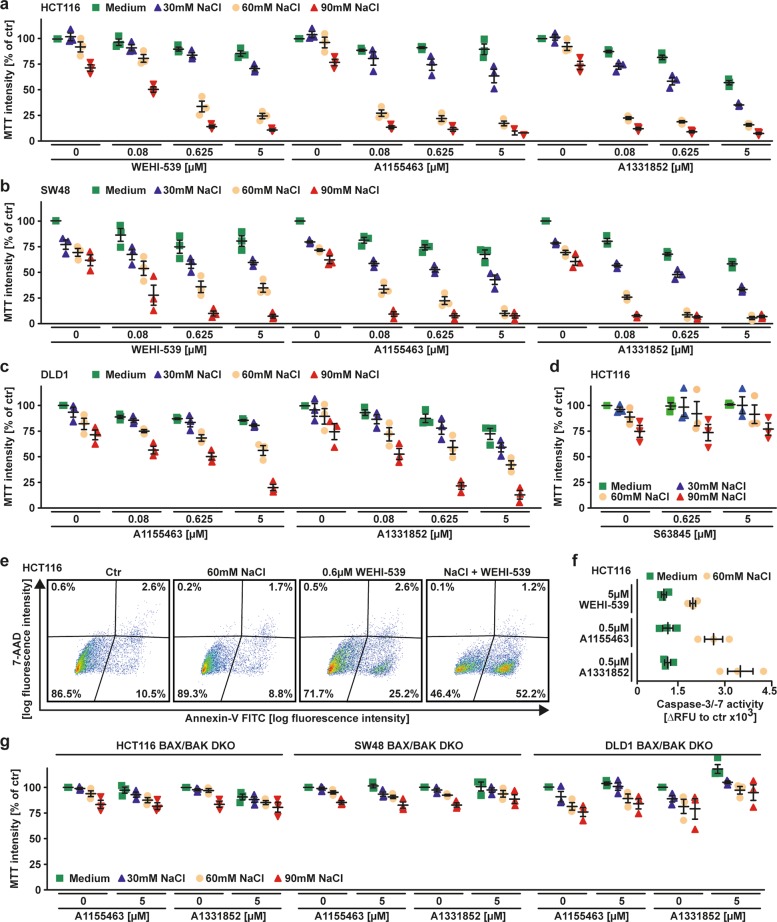
Hyperosmotic stress renders exclusive BCL-XL inhibition sufficient for cell death induction. **a**–**d** Cells were challenged with the indicated concentrations of WEHI-539, A1155463, A1331852 and S63845 for 18 h in the presence and absence of the indicated concentrations of NaCl. **e** HCT116 cells were challenged with WEHI-539 (0.6 µM) and NaCl (60 mM) for 4 h and subsequently analyzed by flow cytometry for 7-AAD- and annexin-V positivity. **f** HCT116 cells were treated with the BCL-XL inhibitors WEHI-539, A1155463 and A1331852 in the presence and absence of NaCl (60 mM). Caspase-3/-7 activity was assessed using the fluorogenic substrate (DEVD)_2_-R110. **g** BAX/BAK-deficient variants of HCT116, DLD1 and SW48 cells were challenged with the indicated concentrations of A1155463 and A1331852 (both BCL-XL selective) for 18 h in the presence and absence of the indicated concentrations of NaCl. For (**a**–**d**, **f** and **g**), data points and mean ± SEM from three independent experiments are shown; for (**e**), data shown are representative of two experiments performed. RFU relative fluorescence units.

In previous work, we showed that hyperosmotic stress facilitates MOMP induction^8–10^. Hyperosmotic stress (or hypertonicity) occurs when osmotically active solutes (such as NaCl) cannot passively diffuse across the plasma membrane and thus establish an osmotic pressure gradient between the extra- and intracellular space. Notably, hypertonicity and BCL-XL-selective BH3 mimetics synergized in cell death induction and abrogated the necessity of concomitant MCL-1 inhibition (Fig. 2a–c, Supplementary Table 2). In contrast, inhibition of MCL-1 was not cytotoxic (Fig. 2d). Targeting BCL-XL under hyperosmotic stress was associated with annexin-V/7-AAD positivity (Fig. 2e) and increased activity of the effector caspases 3 and 7 (Fig. 2f) while loss of BAX/BAK rescued cells from cytotoxic effects (Fig. 2g). Collectively, our data suggested that hyperosmotic stress counteracted the antiapoptotic function of MCL-1 and thereby rendered BCL-XL inhibition sufficient for apoptosis induction. Functionally, combination of BCL-XL inhibitors and hyperosmotic stress resulted in contextual synthetic lethality.

### Hypertonicity-imposed contextual synthetic lethality is selective for BCL-XL-targeting drugs

We assessed whether other drugs also displayed enhanced cytotoxicity under hypertonic conditions and screened a library comprising 110 well-characterized apoptosis-inducing compounds. Hypertonicity only boosted cancer cell killing of BCL-XL targeting drugs such as A1155463, A1331852, ABT-737 and navitoclax (ABT-263), but not other compounds (Supplementary Fig. 1 and Supplementary Tables 3, 4). Hyperosmotic stress apparently granted contextual synthetic lethality specifically to BCL-XL targeting drugs.

### NOXA upregulation is critical for hypertonicity-enforced BCL-XL addiction

**Fig. 3:**
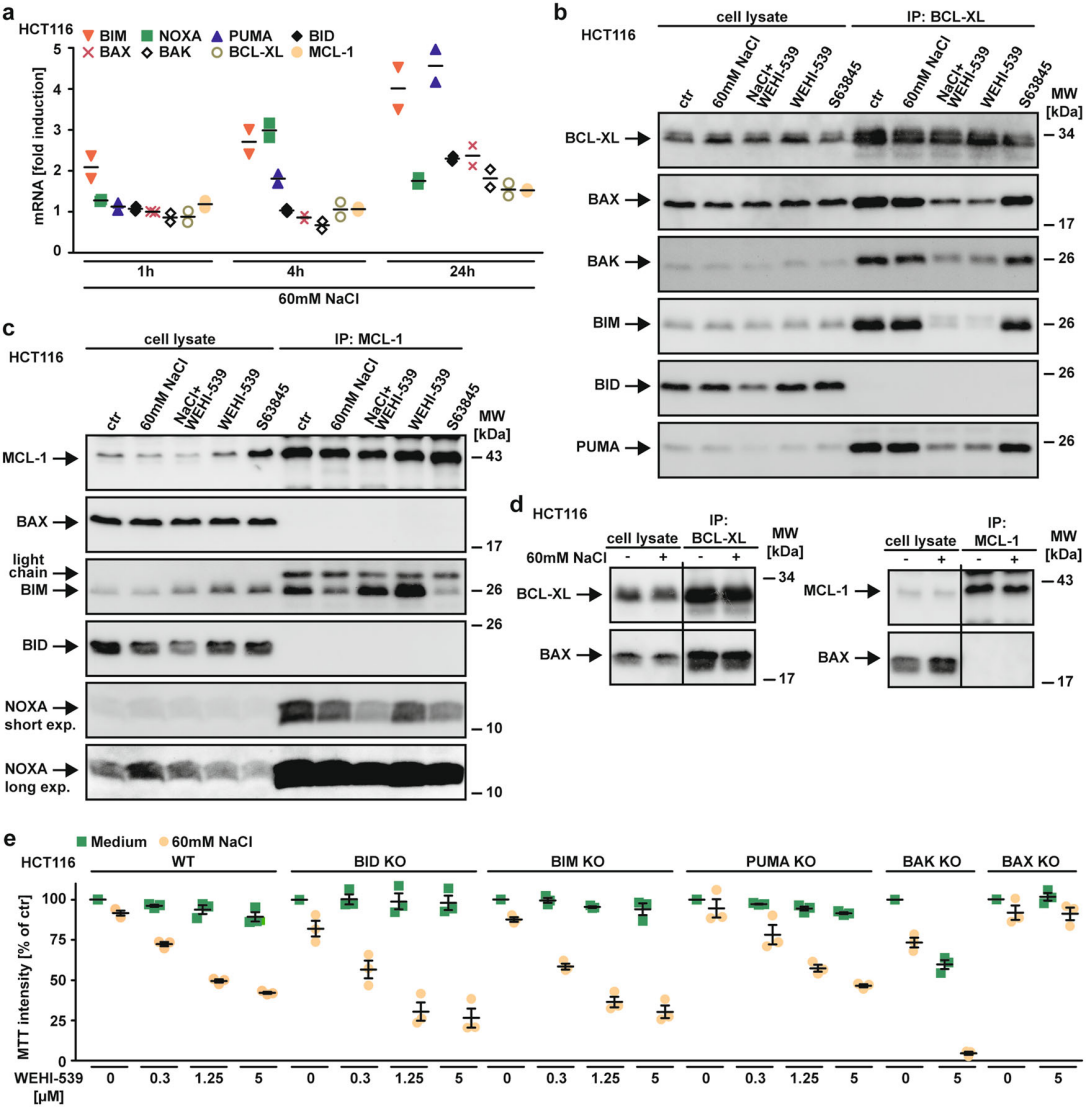
Hypertonicity increases transcription of BCL-2 family proteins without affecting interaction of BCL-XL/MCL-1 with BAX. **a** HCT116 cells were challenged with NaCl (60 mM) for the indicated periods. mRNA levels of genes encoding the indicated proteins were analyzed by qPCR. Shown are data points and mean from two independent experiments. **b**, **c** HCT116 cells were challenged with NaCl (60 mM), WEHI-539 (1.25 µM) and S63845 (2.5 µM) for 5 h. After washing and cell lysis, immunoprecipitation was performed with antibodies specific for **b** BCL-XL and **c** MCL-1. Immunoprecipitates were analyzed together with the corresponding lysates by western blotting using antibodies specific for the indicated proteins. The BCL-XL inhibitor WEHI-539 and the MCL-1 inhibitor S63845 served as positive/negative controls. **d** HCT116 cells were challenged with NaCl (60 mM) for 24 h or left untreated. Immunoprecipitation was performed as described in (**b**). For (**b**–**d**), data shown are representative of at least two experiments performed. **e** HCT116 cells and BID-, BIM-, PUMA-, BAX-, and BAK-deficient variants thereof were challenged with the indicated concentrations of WEHI-539 for 18 h in the presence and absence of NaCl (60 mM). Data points and mean ± SEM from three independent experiments are shown.

**Fig. 4:**
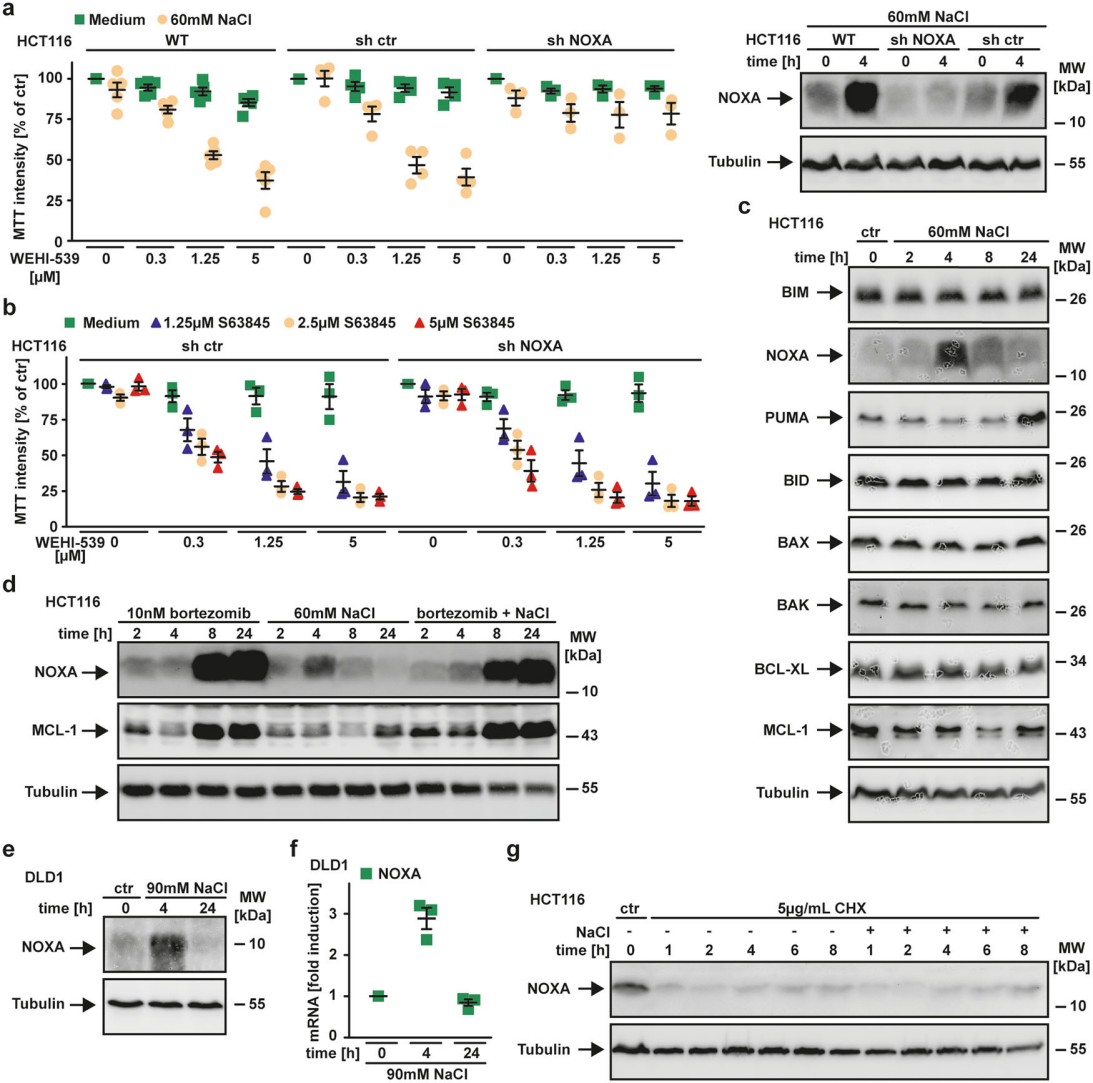
Contextual synthetic lethality of BCL-XL inhibitors under hyperosmotic conditions is NOXA dependent. **a** Left panel: HCT116 cells and NOXA-deficient variants thereof were challenged with the indicated concentrations of WEHI-539 for 18 h in the presence and absence of NaCl (60 mM). Right panel: Western blot analysis of NOXA levels in HCT116, HCT116 shNOXA and HCT116 shControl cells in the presence and absence of NaCl (60 mM). **b** Cells were challenged with the indicated concentrations of WEHI-539 in the presence and absence of the indicated concentrations of the MCL-1 inhibitor S63845 for 18 h. **c**, **e** Cells were challenged with NaCl (HCT116: 60 mM, DLD1: 90 mM) for the indicated periods. After washing and cell lysis, western blot analyses were performed with antibodies specific for the indicated proteins. Detection of tubulin served as a loading control. **d** HCT116 cells were challenged with NaCl (60 mM) in the presence and absence of the proteasome inhibitor bortezomib (10 nM). Changes in cellular NOXA levels were analyzed by western blotting. **f** DLD1 cells were challenged with NaCl (90 mM) for the indicated periods. *PMAIP1* mRNA levels (encoding NOXA) were analyzed by qPCR. **g** HCT116 cells were treated with NaCl (75 mM) for the indicated periods of time in the presence and absence of cycloheximide (CHX, 5 µg/mL), an inhibitor of protein translation. Western blot analysis was performed as in (**c**). For (**a**, **b** and **f**), data points and mean ± SEM from three independent experiments are shown. For (**c**–**e** and **g**), data shown are representative of at least two independent experiments performed.

**Fig. 5:**
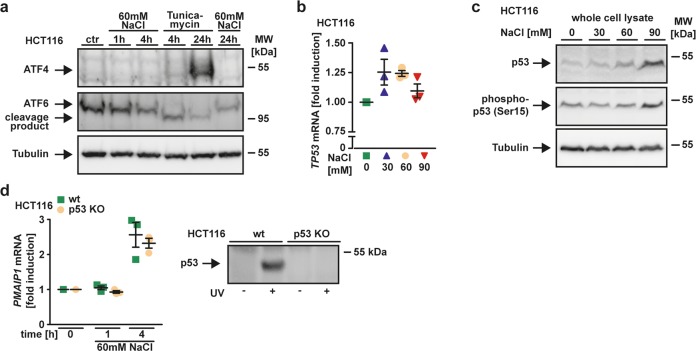
Hypertonicity-induced NOXA upregulation is not related to ER stress and independent of p53. **a** Cells were challenged with NaCl (60 mM) and tunicamycin (2 µg/mL), an inducer of endoplasmic reticulum stress. After washing and cell lysis, western blot analyses were performed with antibodies specific for the indicated proteins. Detection of tubulin served as a loading control. **b** HCT116 cells were challenged with NaCl in the indicated concentrations for 5.5 h. *TP53* mRNA levels were analyzed by qPCR. **c** HCT116 cells were challenged with the indicated concentrations of NaCl for 18 h and subsequently analyzed by western blotting as in (**a**). Hypertonicity-induced phosphorylation of Ser15 indicates functional activation of p53. **d** Left panel: HCT116 cells and p53-deficient variants thereof were challenged with NaCl (60 mM) for the indicated periods. mRNA levels of the NOXA-encoding gene *PMAIP1* were analyzed by qPCR. Right panel: Western blot analysis of p53 levels in UV-treated HCT116 and HCT116 p53 KO cells. For (**a** and **c**), data shown are representative of at least two independent experiments performed. For (**b** and **d**), data points and mean ± SEM from three independent experiments are shown.

We next investigated whether the hypertonicity-induced shift from BCL-XL/MCL-1 codependency to exclusive MCL-1 addiction (Fig. 2a–c) was due to changes in the network of BCL-2 family proteins. We first focused on transcriptional changes under hyperosmotic stress. Exposing HCT116 cells to 60 mM NaCl for 1−4 h caused upregulation of BIM, NOXA and (to a lesser extent) PUMA (Fig. 3a). These early hypertonicity-induced changes did not affect interaction of BCL-XL with BAX and proapoptotic BH3-only proteins (Fig. 3b), but slightly reduced binding of BIM to MCL-1 (Fig. 3c). After 24 h, BIM and PUMA upregulation was even more prominent along with enhanced transcription of BID, MCL-1, BCL-XL, BAX and BAK (Fig. 3a). BAX is essential for MOMP in HCT116 cells and its activation is controlled by interaction with MCL-1 and BCL-XL^5,22,23^. Coimmunoprecipitation experiments, however, failed to demonstrate hypertonicity-induced changes of BCL-XL/MCL-1 interaction with BAX (Fig. 3d). We next investigated the functional relevance of hypertonicity-induced BCL-2 family protein upregulation for exclusive BCL-XL addiction. Genetic loss of the directly BAX-activating proteins BID or BIM failed to rescue NaCl/WEHI-539-treated cells^24,25^ whereas loss of BAX (but not BAK) expectedly did (Fig. 3e). PUMA-deficiency somewhat attenuated NaCl/WEHI-539 cytotoxicity. Surprisingly, deficiency in the MCL-1 interacting BH3-only Protein NOXA granted almost full-blown protection to NaCl/WEHI-539 treatment (Fig. 4a and Supplementary Fig. 2a)^26^. Notably, NOXA-deficient cells still relied on BCL-XL/MCL-1 to safeguard mitochondrial integrity as they were readily killed upon dual BCL-XL/MCL-1 inhibition (Fig. 4b). In line with the transient transcriptional upregulation of NOXA (Figs. 3a and 4f), protein levels peaked approximately 4 h after addition of NaCl and subsequently declined (Fig. 4c, e). This could reflect proteasomal degradation as NOXA is efficiently eliminated via the proteasome and proteasome inhibition consequently caused NOXA accumulation (Fig. 4d)^27^. Hyperosmotic stress did not prolong the half-life of the relatively short-lived NOXA (Fig. 4g), again indicating that the observed transient NOXA accumulation is most probably attributable to increased transcription. Transcriptional upregulation of NOXA has previously been linked to ER stress (with subsequent activation of the transcriptional regulators ATF4 and ATF6) and/or p53^28,29^. However, exposure to 60 mM NaCl for 4 h showed no signs of ATF4 and ATF6 activation (Fig. 5a). Hypertonicity-mediated NOXA-induction also occurred in a p53-independent manner. Addition of NaCl increased p53 mRNA and protein levels (Fig. 5b, c), but p53-deficiency still allowed NOXA upregulation upon NaCl treatment (Fig. 5d). Together, our data demonstrated that transient transcriptional upregulation of NOXA by a not-yet identified mechanism was essential for contextual synthetic lethality of hypertonicity and BCL-XL inhibition.

### Accumulation of NOXA is accompanied by decline of MCL-1 levels

**Fig. 6:**
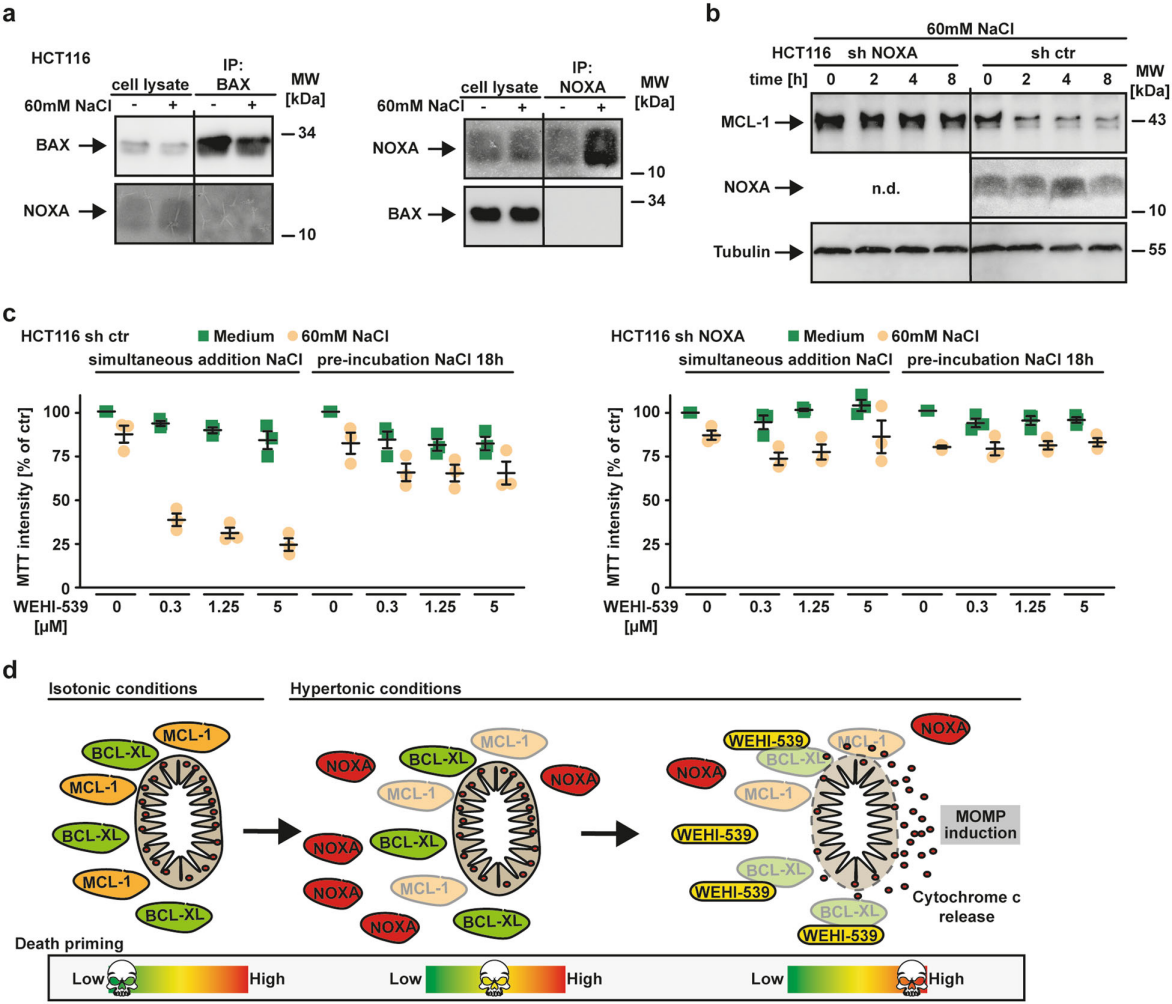
NOXA upregulation and concomitant MCL-1 loss shifts BCL-XL/MCL-1 codependency to exclusive BCL-XL addiction. **a** HCT116 cells were challenged with NaCl (60 mM) for 5 h. After washing and cell lysis, immunoprecipitation was performed with antibodies specific for BAX (left panel) and NOXA (right panel). Immunoprecipitates were analyzed together with the corresponding lysates by western blotting using antibodies specific for the indicated proteins. **b** HCT116 shNOXA and corresponding controls were challenged with NaCl (60 mM) for the indicated periods. MCL-1 and NOXA levels were analyzed by western blotting with antibodies specific for the indicated proteins. **c** Cells were challenged with the indicated concentrations of WEHI-539, either simultaneously with NaCl (60 mM) or after 18 h NaCl preincubation. Viability was assessed by MTT staining. **d** Proposed model of hypertonicity-granted contextual synthetic lethality of BCL-XL inhibition. Left panel: under isotonic conditions, BCL-XL and MCL-1 both safeguard mitochondrial integrity in a functionally redundant and compensatory manner. Due to this dual protection, the cell’s readiness to undergo apoptosis (“death priming”) is low. Middle panel: Hyperosmotic stress triggers transient upregulation of NOXA and temporary decline in MCL-1 levels. Functionally, this generates exclusive BCL-XL-addiction for a limited period. Right panel: Targeting BCL-XL during the time of exclusive BCL-XL dependency irrevocably triggers mitochondrial outer membrane permeabilization (MOMP) and is contextually synthetically lethal. n.d. not detected.

Thus far, we demonstrated that hypertonicity (a) facilitated MOMP induction, (b) shrank dual BCL-XL/MCL-1 protection to exclusive BCL-XL addiction and (c) triggered upregulation of NOXA, a MCL-1 interacting BH3-only protein. We next assessed the interrelations of these observations. NOXA is capable to facilitate or induce MOMP through direct interaction with and activation of BAX or targeting MCL-1 for proteasomal degradation^30–32^. Coimmunoprecipitation experiments did not point to a direct NOXA/BAX interaction during hyperosmotic stress (Fig. 6a). However, hypertonicity-induced NOXA upregulation was followed by a decline in MCL-1 levels that recovered when NOXA expression at later time points returned to baseline (Fig. 4c). NOXA can interact with and target MCL-1 for proteasomal degradation^33–36^. Indeed, we observed that NOXA-deficiency significantly impaired decrease of MCL-1 levels under hyperosmotic stress (Fig. 6b). However, MCL-1 levels started to decline as early as 2 h after exposure to NaCl (Fig. 6b and Supplementary Fig. 2b), whereas NOXA upregulation was only detectable after 4 h (Fig. 6b). Additionally, coimmunoprecipitation experiments showed reduced (rather than the expected enhanced) binding of NOXA to MCL-1 under hypertonic conditions (Fig. 3c). These observations suggested that mechanisms other than NOXA upregulation (e.g., translational repression^37^) might account for or contribute to loss of MCL-1 during hyperosmotic stress. As hypertonicity-induced NOXA upregulation peaked approximately 4 h after addition of NaCl and subsequently declined (Fig. 4c), NOXA-mediated contextual synthetic lethality of hyperosmotic stress and BCL-XL inhibitors should depend on the timing of hypertonicity-induction and BCL-XL inhibition. Indeed, NOXA-proficient cells displayed enhanced WEHI-539 cytotoxicity upon simultaneous NaCl/WEHI-539 treatment. However, preincubation with NaCl for 18 h allowed re-adjustment of NOXA levels to baseline (Fig. 4c, e) and BCL-XL inhibition was consequently not cytotoxic (Fig. 6c). NOXA-deficiency expectedly protected HCT116 cells from WEHI-539-mediated cytotoxicity in presence of NaCl. Our data thus suggested that hyperosmotic stress temporarily and inversely affected cellular levels of MCL-1 and NOXA. Functionally, this resulted in transient exclusive BCL-XL dependency.

## Discussion

Our study demonstrates that hypertonicity-induced, transient upregulation of NOXA counteracts the antiapoptotic function of MCL-1 and temporarily renders dually BCL-XL/MCL-1-protected cells exclusively BCL-XL-dependent. During this period, concomitant targeting of BCL-XL is sufficient to initiate MOMP (Fig. 6d). Functionally, we demonstrate contextual synthetic lethality of BCL-XL inhibitors in an exogenously modified, hypertonic tumor environment (“osmotic reprogramming”) and provide insight into the underlying molecular mechanism that critically involves NOXA. This BH3-only protein determines sensitivity to BCL-XL targeting BH3 mimetics in various cancer entities^32,38–41^. In previous work, we showed that hyperosmotic stress is capable to facilitate activation of the intrinsic apoptosis pathway^8–10,42^. However, in these studies we repeatedly failed to detect hypertonicity-induced disturbances in the BCL-2 protein family network. Retrospectively, this was for the simple reason that we left early, transient changes out of consideration. Instead, we looked for alterations after 18 or 24 h of NaCl treatment, when cytotoxic effects of BCL-XL inhibitor became apparent. Our reasoning that hypertonicity-induced changes in cellular levels of BCL-2 family proteins should be observable at the time of measurable cytotoxicity proved false. The current study highlights that hypertonicity-induced upregulation of NOXA and decline in MCL-1 levels are both transient events. Consequently, exclusive BCL-XL addiction and contextual synthetic lethality of BCL-XL inhibitors is restricted to a certain window (Fig. 6d). A transient decrease in cellular MCL-1 levels under hypertonic conditions was already observed earlier^37^. We also noticed that (in contrast to NOXA) hypertonicity-induced upregulation of BIM-encoding mRNA (Fig. 3a) did not translate into increased protein levels (Fig. 3b,c). This could reflect translational repression and/or changes mRNA stability^37,43^. Interestingly, BCL-XL inhibitors induced apoptosis under hyperosmotic conditions in the absence of BIM (Fig. 3e). A previous study, however, convincingly demonstrated that NOXA-triggered release of BIM from MCL-1 was essential for BAX/BAK activation^44^. Our results resemble findings from another study that showed dispensability of BIM (and other BH3-only proteins) for apoptosis induction when MCL-1 and BCL-XL are inhibited^5^. Whether attenuated cytotoxicity of NaCl/WEHI-539 in PUMA-deficient cells indicates involvement of PUMA in hypertonicity-enforced BCL-XL addiction requires further studies (Fig. 3e).

Biophysical factors in the environment of tumors (such as low oxygen levels or elevated pressure) enforce adaption of cancer cells to ensure survival. The resulting cellular processes have thus far mainly been recognized as drivers of non-cell-autonomous resistance to treatment^45^. Vice versa, interfering with these environment-imposed adaptive cellular responses is often lethal for cancer cells and is known as contextual synthetic lethality^46^. Indeed, environment-based contextual synthetic lethality has been recognized as potential therapeutic target^46–48^. Our current study advances the concept of contextual synthetic lethality inasmuch as we exogenously modified biophysical properties in the tumor environment (“osmotic reprogramming”) in a way that allowed hijacking a cancer cell’s adaptive response. Therapeutic exploitation of our findings has of course limitations. For practical reasons, artificial generation of hyperosmotic stress around tumors is conceivable in solid cancers that are easily reachable, but hardly for e.g. leukemia. Notably, our data indicate that exposure of cancer cells to osmotically active solutes for just a couple of hours is sufficient to promote NOXA upregulation (Figs. 3a and 4c). Even transient hypertonicity could therefore be sufficient to render concomitant BCL-XL inhibition deleterious for cancer cells. Moreover, contextual synthetic lethality of hyperosmotic stress and selective inhibition of BCL-2-like proteins might only be achievable in cells that are (a) capable to upregulate NOXA and (b) exhibit a codependency that can be targeted by NOXA (e.g. MCL-1 or BFL-1). While we found clear evidence for hypertonicity-induced, transient transcriptional upregulation of NOXA (Fig. 3a), the underlying signaling cascades and transcription factor(s) involved remain to be determined. Our approach additionally requires codependency on BCL-2-like proteins that are druggable. There are to date no selective inhibitors for BCL-W, which can render cancer cells nonresponsive to BCL-XL and MCL-1 inhibition^4^. However, tools to individually decipher the dependencies of cancer cells on BCL-2-like proteins are available and allow (at least to some extent) to predict treatment response beforehand^2,49^. Nevertheless, our study provides evidence that reprogramming biophysical factors in the tumor environment can alter dependency of cancer cells on BCL-2-like proteins and thereby grant contextual synthetic lethality to BH3 mimetics.

## Material and methods

### Cell lines, antibodies and reagents

HCT116 cells were obtained from the German Collection of Microorganisms and Cell Culture (DSMZ, Braunschweig, Germany). HCT116 BAX/BAK DKO and HCT116 BAK KO cells were kindly provided by Richard Youle (National Institutes of Health, Bethesda, USA)^22^. HCT116 BAX KO cells were obtained from Bert Vogelstein (Johns Hopkins University, Baltimore, MA, USA)^50^, BID-deficient HCT116 cells were a kind gift from Xu Luo (University of Nebraska Medical Center, Nebraska, USA)^51^, HCT116 BIM KO cells were provided by Hamsa Puthalakath (La Trobe University, Bundoora, Australia)^52^, HCT116 shNOXA and HCT116 shCtr cells have been described previously^53^. HCT116 PUMA KO cells were purchased from Horizon Discovery (Cambridge, UK)^54^. SW48 and DLD1 cells and BAX/BAK-deficient variants thereof were purchased from Sigma (Steinheim, Germany). All cell lines were maintained in RPMI 1640 medium (PAN Biotech, Aidenbach, Germany) with 10% (v/v) fetal calf serum (Sigma). Antibodies: ATF4 (#11815), ATF6 (#65880), BID (#2002), BIM (#2933), BAX (#5023), BAK (#12105), PUMA (#12450), BCL-XL (#2764), p53 (#4866); phospho-p53 (#9284) Cell Signaling (Beverly, MA, USA); MCL-1 (ab32087): abcam (Cambridge, UK); tubulin (#MS-581): Dunnlab (Asbach, Germany); NOXA (114C307, #sc-56169): Santa Cruz (Santa Cruz, CA, USA). Chemicals: MTT (3-[4,5-dimethylthiazol-2-yl]-2,5-diphenyl tetrazolium bromide), cycloheximide: Biomol (Hamburg, Germany); A1155463, A1331852, ABT-199, ABT-737, S63845, WEHI-539 and apoptosis compound library (HY-L003): Hycultec (Beutelsbach, Germany).

### MTT-based cell viability assay

Cells were seeded in 96-well plates (2 × 10^4^ cells/well) and challenged with the indicated concentrations of the indicated substances in duplicates (technical replicates). Unless indicated otherwise, cell viability was determined 18 h after stimulation using MTT staining (2 h at 37 °C). Staining intensity was measured at 595 nm and the mean was calculated from the technical replicates of each experiment. The mean value for untreated controls was set to 100%. For any other condition, the MTT staining intensity is given relative to the corresponding untreated group (% of control). Data points shown are mean values (calculated from two technical replicates) of independent experiments (*n* ≥ 2).

### Western blot analysis

Cells were harvested by centrifugation and lysed in 4× Laemmli sample buffer (8% [w/v] SDS, 0.1 M dithiothreitol, 40% [v/v] glycerol, 0.2 M Tris, pH 8.0) supplemented with phosphatase inhibitor cocktails-I and -II (Sigma). Samples were sonicated and boiled for 5 min at 96 °C before proteins were separated by SDS-PAGE and transferred to polyvinylidene difluoride membranes. To block nonspecific binding sites, membranes were incubated in Tris-buffered saline containing 0.1% (v/v) Tween 20 and 5% (w/v) dry milk before primary antibodies of the specificity of interest were added. Antigen−antibody complexes were visualized using horseradish peroxidase-conjugated secondary antibodies (Dako, Hamburg, Germany) and ECL technology (Pierce, Rockford, IL, USA).

### Coimmunoprecipitation

HCT116 cells (8 × 10^6^ cells per condition) were challenged with the indicated substances for the indicated periods. After washing with ice-cold PBS, cell were lysed in MCBL-buffer (NP40 0.5% [v/v], 150 mM NaCl, 50 mM Tris, pH 7.4) supplemented with complete^TM^ protease inhibitor cocktail (Roche, Mannheim, Germany) for 40 min on ice. Lysates were cleared by centrifugation (20,000 × *g*, 20 min, 4 °C) and incubated overnight with a 1:200 dilution of BCL-XL-, MCL-1-, BAX- and NOXA-specific antibodies at 4 °C. The next day, antigen–antibody complexes were precipitated using protein G agarose (Roche). After washing in lysis buffer, agarose-bound proteins were eluted by incubation at 95 °C in Laemmli sample buffer (10 min) and analyzed together with the corresponding lysates by western blotting.

### Quantitative PCR

Total RNAs were isolated with the RNeasy mini kit (Qiagen, Valencia, CA, USA), according to the manufacturer’s instructions. Two micrograms of total RNA was transcribed into complementary DNA using the high-capacity cDNA reverse transcription kit (Applied Biosystems, Carlsbad, CA, USA). Quantification of mRNA levels of the following genes was done using TaqMan gene expression assays (Applied Biosystems) and an ABI Prism 7900 sequence detector (Applied Biosystems): *PMAIP1* (#Hs00560402_m1), *BCL2L1* (#Hs00236329_m1), *BAK1* (#Hs00832846_g1), *BBC3* (#Hs00248075_m1), *MCL1* (#Hs01050896_m1), *BCL2L11* (#Hs01076940_m1), *BID* (#Hs00609632_m1), *BAX* (#Hs00180269_m1), *TP53* (#Hs01034249_m1). qRT-PCR reactions were performed in triplicates for each sample and were normalized to the expression of the housekeeping gene *HPRT1* (Hs02800695_m1). mRNA levels were calculated using the SDS 2.1 software (Applied Biosystems).

### Flow cytometry

Cell death was assessed by annexin-V and 7-aminoactinomycin D (7-AAD) staining. In brief, HCT116 cells were challenged with WEHI-539 and S63845 for 24 h or left untreated. Afterwards, cells were stained with 7-AAD and annexin-V (4 °C for 15 min in the dark) and analyzed immediately using a FACSCanto flow cytometer (BD Biosciences, Heidelberg, Germany) following standard procedures^55^.

## Supplementary information

Supplementary Figure and Table Legends

Supplementary Figure 1

Supplementary Figure 2

Supplementary Table 1

Supplementary Table 2

Supplementary Table 3

Supplementary Table 4
