# Supplementary material for: NOXA-dependent contextual synthetic lethality of BCL-XL inhibition and “osmotic reprogramming” in colorectal cancer
Source: Cell Death Dis. 2020 Apr 20;11(4):257. doi: 10.1038/s41419-020-2446-8 (PMC7171071; doi:10.1038/s41419-020-2446-8)
Supplement: Supplementary file 1 — Supplementary Figure and Table Legends [file 41419_2020_2446_MOESM1_ESM.doc]

## Supplementary figure and table legends

## Supplementary Figure 1: Hyperosmotic stress selectively enhances cytotoxicity of BCL‑XL-targeting drugs

HCT116 cells were challenged with the indicated compounds in concentrations ranging from 0 to 20 µM in the presence and absence of NaCl (60 mM) for 18 h. Shown are mean values from three independent experiments. For detailed information on compounds, please see Supplementary Table 3.

## Supplementary Figure 2: Hyperosmotic stress decreases MCL‑1 levels in DLD1 cells

(a) DLD1 cells were transfected with 100 nM siRNA oligonucleotides targeting NOXA (ON-TARGETplus SMARTpool #L-005275-00-005, Dharmacon, Lafayette, CO, USA) or non-targeting control (#D-001810-10-05, Dharmacon) using DharmaFECT 1 transfection reagent (#T-2001-02, Dharmacon) according to manufacturer’s instructions. After 48 h, cells were challenged with the BCL‑XL selective BH3 mimetic A1155463 (1.25 µM) in the presence and absence of NaCl (90 mM) for another 18 h. Shown are data points and mean from two independent experiments. (b) DLD1 cells were challenged with NaCl (90 mM) for the indicated periods. MCL‑1 levels were analyzed by western blotting. Detection of tubulin served as loading control. Data shown are representative of two experiments performed.

## Supplementary Table 1: Combination Index of BCL‑XL/MCL‑1 co-inhibition

Synergistic cell death induction in HCT116 cells by combinatorial treatment with S63845 and ABT‑737, WEHI‑539, ABT‑199, A1155463 or A1331852 was quantitatively assessed by calculating the combination index (CI). CI values <1 are considered to be synergistic and CI > 1 indicate antagonistic effects. Strength of synergism can be further graded: <0.1 very strong synergism, 0.1–0.3 strong synergism, 0.3–0.7 synergism, 0.7–0.85 moderate synergism, 0.85–0.9 slight synergism, 0.9–1.1 no synergism but nearly additive effects. CI values were calculated from the mean values of three independent experiments with the freely available software CompuSyn version 1.0 using the median effect/combination index isobologram method[^1^](#_ENREF_1).

**Reference:**

1. Chou, T. C. Theoretical basis, experimental design, and computerized simulation of synergism and antagonism in drug combination studies*. Pharmacol. Re*v**. 5**8, 621-681 (2006).

## Supplementary Table 2: Combination Index of BCL‑XL-targeting BH3 mimetics and hyperosmotic stress

Synergistic cell death induction in HCT116, SW48 and DLD1 cells by combinatorial treatment with NaCl plus WEHI‑539, A1155463 or A1331852 was quantitatively assessed by calculating the combination index (CI) as above. Again, CI values <1 indicate synergism, whereas CI > 1 indicate antagonistic effects. CI values were calculated from mean values of three independent experiments.

## Supplementary Table 3: Apoptosis compound library

Information on compounds included in the screening library (apoptosis compound library #HY-L003, Hycultec, Beutelsbach, Germany). Compounds that displayed strongly enhanced cytotocixity under hyperosmotic stress are highlighted.

## Supplementary Table 4: IC_50_ values of cytotoxic compounds

For compounds that displayed cytotoxic activity (please see Supplementary Figure 1), IC_50_ values and the 95% confidence interval in the presence and absence of NaCl were calculated (non-linear regression analysis; inhibitor vs. normalized response, variable slope) using GrapPad Prism7 (GraphPad Software, San Diego, CA, USA). Values shown were calculated from at least three independent experiments. Compounds that displayed strongly enhanced cytotoxicity under hyperosmotic stress are highlighted. Dashed lines indicate that the tested compound exerted no cytotoxic effect.
