# Supplementary figures and images for: NOXA-dependent contextual synthetic lethality of BCL-XL inhibition and “osmotic reprogramming” in colorectal cancer
Source: Cell Death Dis. 2020 Apr 20;11(4):257. doi: 10.1038/s41419-020-2446-8 (PMC7171071; doi:10.1038/s41419-020-2446-8)

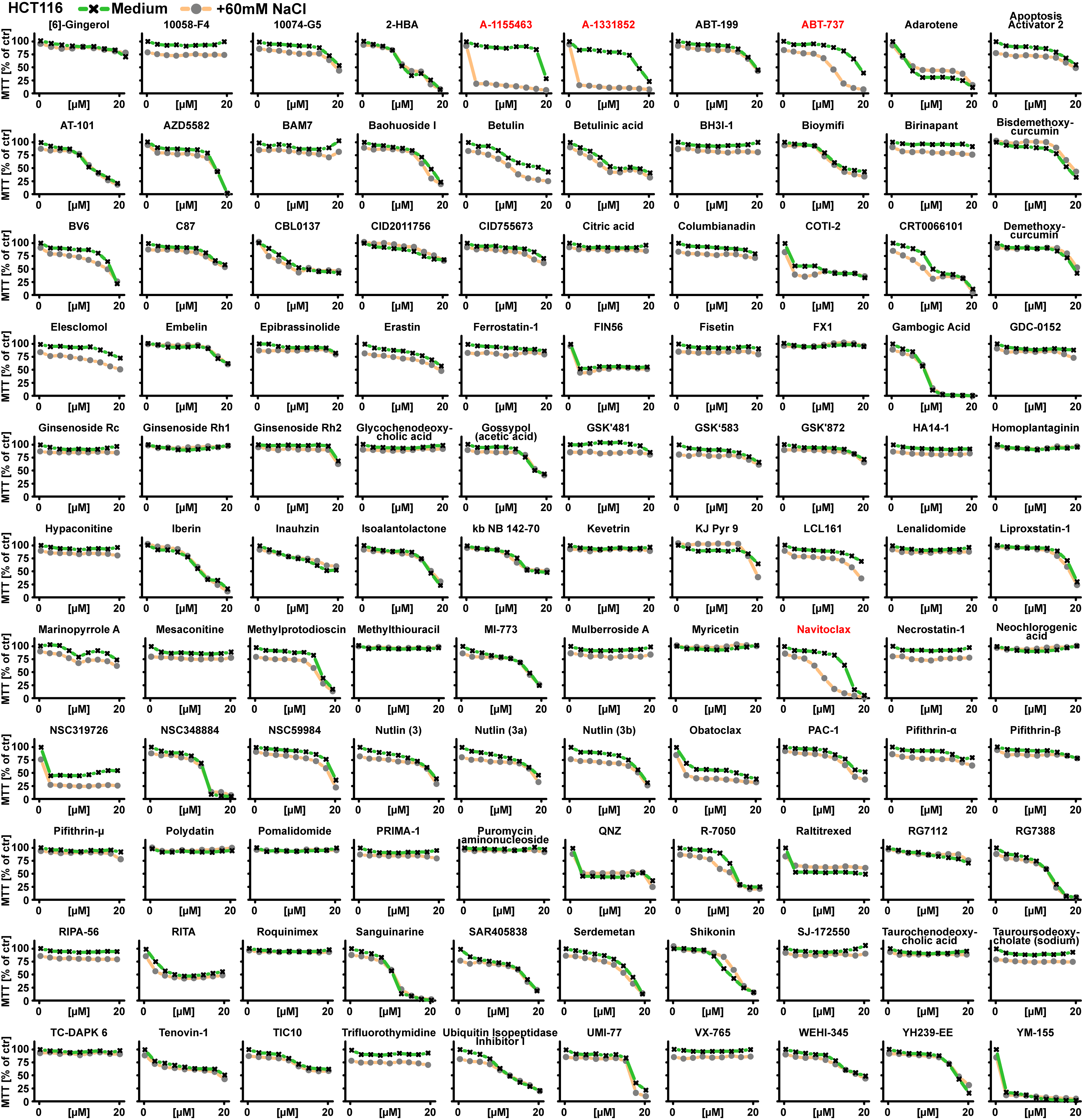

Supplement: Supplementary file 2 — Supplementary Figure 1 [file 41419_2020_2446_MOESM2_ESM.tif]

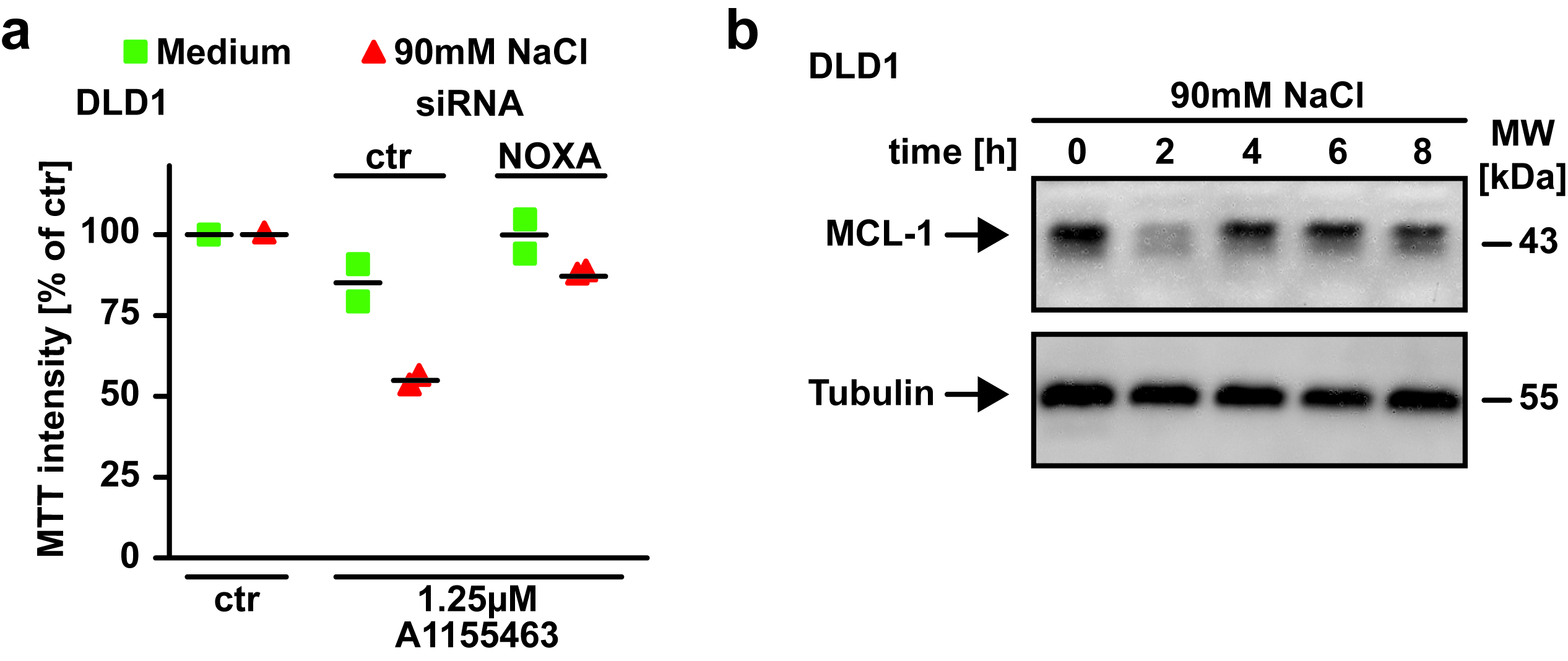

Supplement: Supplementary file 3 — Supplementary Figure 2 [file 41419_2020_2446_MOESM3_ESM.tif]
