# Supplementary material for: NOXA-dependent contextual synthetic lethality of BCL-XL inhibition and “osmotic reprogramming” in colorectal cancer
Source: Cell Death Dis. 2020 Apr 20;11(4):257. doi: 10.1038/s41419-020-2446-8 (PMC7171071; doi:10.1038/s41419-020-2446-8)
Supplement: Supplementary file 4 — Supplementary Table 1 [file 41419_2020_2446_MOESM4_ESM.doc]

## Supplementary Table 1: Combination Index of BCL‑XL/MCL‑1 co-inhibition

| **HCT116** |  | **S63845 [µM]** | | |
| --- | --- | --- | --- | --- |
|  |  | **1.25** | **2.5** | **5** |
| **ABT-737 [µM]** | **0.3** | 0.0892 | 0.0933 | 0.0344 |
|  | **1.25** | 0.0583 | 0.0404 | 0.0211 |
|  | **5** | 0.0433 | 0.0390 | 0.0411 |
| **WEHI-539 [µM]** | **0.3** | 0.0098 | 0.0063 | 0.0058 |
|  | **1.25** | 0.0027 | 0.0021 | 0.0025 |
|  | **5** | 0.0066 | 0.0063 | 0.0070 |
| **­ABT-199 [µM]** | **0.3** | 0.3204 | 0.9511 | 1.3126 |
|  | **1.25** | 0.5794 | 0.7616 | 0.7287 |
|  | **5** | 0.5125 | 0.5459 | 0.5210 |
| **A1155463 [µM]** | **0.3** | 3.46E-5 | 4.77E-5 | 8.66E-5 |
|  | **1.25** | 1.80E-5 | 2.91E-5 | 6.45E-5 |
|  | **5** | 1.34E-5 | 2.36E-5 | 5.25E-5 |
| **A1331852 [µM]** | **0.3** | 0.0303 | 0.0286 | 0.0308 |
|  | **1.25** | 0.1066 | 0.1067 | 0.1070 |
|  | **5** | 0.3727 | 0.3729 | 0.3731 |
