# Supplementary material for: NOXA-dependent contextual synthetic lethality of BCL-XL inhibition and “osmotic reprogramming” in colorectal cancer
Source: Cell Death Dis. 2020 Apr 20;11(4):257. doi: 10.1038/s41419-020-2446-8 (PMC7171071; doi:10.1038/s41419-020-2446-8)
Supplement: Supplementary file 5 — Supplementary Table 2 [file 41419_2020_2446_MOESM5_ESM.doc]

## Supplementary Table 2: Combination Index of BCL‑XL-targeting BH3 mimetics and hyperosmotic stress

| **HCT116** |  | **NaCl [mM]** | | |
| --- | --- | --- | --- | --- |
|  |  | **30** | **60** | **90** |
| **WEHI-539 [µM]** | **0.08** | 0.3745 | 0.1310 | 0.0129 |
|  | **0.625** | 0.3248 | 0.0095 | 0.0019 |
|  | **5** | 0.6416 | 0.0332 | 0.0076 |
| **A1155463 [µM]** | **0.08** | 0.0237 | 5.33E-4 | 1.49E-4 |
|  | **0.625** | 0.1217 | 0.0031 | 0.0010 |
|  | **5** | 0.4326 | 0.0152 | 0.0040 |
| **A1331852 [µM]** | **0.08** | 0.0619 | 0.0039 | 0.0014 |
|  | **0.625** | 0.2373 | 0.0257 | 0.0087 |
|  | **5** | 0.5823 | 0.1586 | 0.0594 |

| **SW48** |  | **NaCl [mM]** | | |
| --- | --- | --- | --- | --- |
|  |  | **30** | **60** | **90** |
| **WEHI-539 [µM]** | **0.08** | 0.4719 | 0.3900 | 0.1019 |
|  | **0.625** | 0.3440 | 0.1448 | 0.0161 |
|  | **5** | 1.0935 | 0.3004 | 0.0172 |
| **A1155463 [µM]** | **0.08** | 0.2456 | 0.0918 | 0.0119 |
|  | **0.625** | 0.3106 | 0.0571 | 0.0124 |
|  | **5** | 0.8241 | 0.0621 | 0.0467 |
| **A1331852 [µM]** | **0.08** | 0.2594 | 0.0614 | 0.0102 |
|  | **0.625** | 0.3330 | 0.0194 | 0.0157 |
|  | **5** | 0.7920 | 0.0460 | 0.0754 |

| **DLD1** |  | **NaCl [mM]** | | |
| --- | --- | --- | --- | --- |
|  |  | **30** | **60** | **90** |
| **A1155463 [µM]** | **0.08** | 0.7863 | 0.4396 | 0.1474 |
|  | **0.625** | 0.8323 | 0.3265 | 0.1235 |
|  | **5** | 2.6036 | 0.4401 | 0.0367 |
| **A1331852 [µM]** | **0.08** | 0.4480 | 0.1464 | 0.0478 |
|  | **0.625** | 0.4616 | 0.1547 | 0.0146 |
|  | **5** | 0.9038 | 0.3412 | 0.0400 |
