# Supplementary material for: NOXA-dependent contextual synthetic lethality of BCL-XL inhibition and “osmotic reprogramming” in colorectal cancer
Source: Cell Death Dis. 2020 Apr 20;11(4):257. doi: 10.1038/s41419-020-2446-8 (PMC7171071; doi:10.1038/s41419-020-2446-8)
Supplement: Supplementary file 6 — Supplementary Table 3 [file 41419_2020_2446_MOESM6_ESM.doc]

## Supplementary Table 3: Apoptosis compound library

| **Compound** | **CAS number** |
| --- | --- |
| [6]-Gingerol | 23513-14-6 |
| 10058-F4 | 403811-55-2 |
| 10074-G5 | 413611-93-5 |
| 2-HBA | 131359-24-5 |
| **A-1155463** | **1235034-55-5** |
| **A-1331852** | **1430844-80-6** |
| ABT-199 | 1257044-40-8 |
| **ABT-737** | **852808-04-9** |
| Adarotene | 496868-77-0 |
| Apoptosis Activator 2 | 79183-19-0 |
| AT-101 | 866541-93-7 |
| AZD5582 | 1258392-53-8 |
| BAM7 | 331244-89-4 |
| Baohuoside I | 113558-15-9 |
| Betulin | 473-98-3 |
| Betulinic acid | 472-15-1 |
| BH3I-1 | 300817-68-9 |
| Bioymifi | 1420071-30-2 |
| Birinapant | 1260251-31-7 |
| Bisdemethoxycurcumin | 33171-05-0 |
| BV6 | 1001600-56-1 |
| C87 | 332420-90-3 |
| CBL0137 | 1197397-89-9 |
| CID2011756 | 638156-11-3 |
| CID755673 | 521937-07-5 |
| Citric acid | 77-92-9 |
| Columbianadin | 5058-13-9 |
| COTI-2 | 1039455-84-9 |
| CRT0066101 | 1883545-60-5 |
| Demethoxycurcumin | 22608-11-3 |
| Elesclomol | 488832-69-5 |
| Embelin | 550-24-3 |
| Epibrassinolide | 78821-43-9 |
| Erastin | 571203-78-6 |
| Ferrostatin-1 | 347174-05-4 |
| FIN56 | 1083162-61-1 |
| Fisetin | 528-48-3 |
| FX1 | 1426138-42-2 |
| Gambogic Acid | 2752-65-0 |
| GDC-0152 | 873652-48-3 |
| Ginsenoside Rc | 11021-14-0 |
| Ginsenoside Rh1 | 63223-86-9 |
| Ginsenoside Rh2 | 78214-33-2 |
| Glycochenodeoxycholic acid | 640-79-9 |
| Gossypol (acetic acid) | 12542-36-8 |
| GSK’583 | 1346547-00-9 |
| GSK'481 | 1622849-58-4 |
| GSK'872 | 1346546-69-7 |
| HA14-1 | 65673-63-4 |
| Homoplantaginin | 17680-84-1 |
| Hypaconitine | 6900-87-4 |
| Iberin | 505-44-2 |
| Inauhzin | 309271-94-1 |
| Isoalantolactone | 470-17-7 |
| kb NB 142-70 | 1233533-04-4 |
| Kevetrin (hydrochloride) | 66592-89-0 |
| KJ Pyr 9 | 581073-80-5 |
| LCL161 | 1005342-46-0 |
| Lenalidomide | 191732-72-6 |
| Liproxstatin-1 | 950455-15-9 |
| Marinopyrrole A | 1227962-62-0 |
| Mesaconitine | 2752-64-9 |
| Methylprotodioscin | 54522-52-0 |
| Methylthiouracil | 56-04-2 |
| MI-773 | 1303607-07-9 |
| Mulberroside A | 102841-42-9 |
| Myricetin | 529-44-2 |
| **Navitoclax** | **923564-51-6** |
| Necrostatin-1 | 4311-88-0 |
| Neochlorogenic acid | 906-33-2 |
| NSC319726 | 71555-25-4 |
| NSC348884 | 81624-55-7 |
| NSC59984 | 803647-40-7 |
| Nutlin (3) | 548472-68-0 |
| Nutlin (3a) | 675576-98-4 |
| Nutlin (3b) | 675576-97-3 |
| Obatoclax | 803712-79-0 |
| PAC-1 | 315183-21-2 |
| Pifithrin-α (hydrobromide) | 63208-82-2 |
| Pifithrin-β (hydrobromide) | 511296-88-1 |
| Pifithrin-μ | 64984-31-2 |
| Polydatin | 27208-80-6 |
| Pomalidomide | 19171-19-8 |
| PRIMA-1 | 5608-24-2 |
| Puromycin aminonucleoside | 58-60-6 |
| QNZ | 545380-34-5 |
| R-7050 | 303997-35-5 |
| Raltitrexed | 112887-68-0 |
| RG7112 | 939981-39-2 |
| RG7388 | 1229705-06-9 |
| RIPA-56 | 1956370-21-0 |
| RITA | 213261-59-7 |
| Roquinimex | 84088-42-6 |
| Sanguinarine | 5578-73-4 |
| SAR405838 | 1303607-60-4 |
| Serdemetan | 881202-45-5 |
| Shikonin | 517-89-5 |
| SJ-172550 | 431979-47-4 |
| Taurochenodeoxycholic acid | 516-35-8 |
| Tauroursodeoxycholate (sodium) | 35807-85-3 |
| TC-DAPK 6 | 315694-89-4 |
| Tenovin-1 | 380315-80-0 |
| TIC10 | 1616632-77-9 |
| Trifluorothymidine | 70-00-8 |
| Ubiquitin Isopeptidase Inhibitor I, | 108477-18-5 |
| UMI-77 | 518303-20-3 |
| VX-765 | 273404-37-8 |
| WEHI-345 | 1354825-58-3 |
| YH239-EE | 1364488-67-4 |
| YM-155 | 781661-94-7 |
