# Supplementary material for: NOXA-dependent contextual synthetic lethality of BCL-XL inhibition and “osmotic reprogramming” in colorectal cancer
Source: Cell Death Dis. 2020 Apr 20;11(4):257. doi: 10.1038/s41419-020-2446-8 (PMC7171071; doi:10.1038/s41419-020-2446-8)
Supplement: Supplementary file 7 — Supplementary Table 4 [file 41419_2020_2446_MOESM7_ESM.doc]

## Supplementary Table 4: IC_50_ values of cytotoxic compounds

| Compound | IC_50 Medium_ [µM] | IC_50 NaCl_ [µM] | 95% CI _Medium_ [µM] | 95% CI _NaCl_ [µM] |
| --- | --- | --- | --- | --- |
| [6]-Gingerol | 2,77E+02 | 1,38E+05 | 1,47E+00 to 2,19E+00 | 3,90E+02 to +∞ |
| 10058-F4 | --- | --- | --- | --- |
| 10074-G5 | 2,56E+01 | 3,48E+01 | 2,09E+01 to 3,45E+01 | 1,77E+01 to 1,23E+02 |
| 2-HBA | 1,79E+00 | 1,98E+00 | 1,47E+00 to 2,19E+00 | 1,62E+00 to 2,42E+00 |
| **A-1155463** | 1,60E+01 | 6,35E-04 | 1,40E+01 to 1,97E+01 | 8,36E-08 to 9,43E-03 |
| **A-1331852** | 8,81E+00 | 1,28E-05 | 6,91E+00 to 1,18E+01 | 3,13E-17 to 1,54E-03 |
| ABT-199 | 1,83E+01 | 2,52E+01 | 1,52E+01 to 2,42E+01 | 1,36E+01 to 9,38E+01 |
| **ABT-737** | 1,81E+01 | 1,46E+00 | 1,47E+01 to 2,42E+01 | 1,18E+00 to 1,80E+00 |
| Adarotene | 3,34E-01 | 8,38E-01 | 1,81E-01 to 5,16E-01 | 0,46E+00 to 1,35E+00 |
| Apoptosis Activator 2 | 3,62E+01 | 6,63E+01 | 2,32E+01 to 7,40E+01 | 1,65E+01 to 8,79E+03 |
| AT-101 | 3,41E+00 | 3,14 E+00 | 3,10E+00 to 3,75E+00 | 2,55E+00 to 3,86E+00 |
| AZD5582 | 8,10E+00 | 5,47 E+00 | 6,62E+00 to 9,67E+00 | 3,59E+00 to 8,49E+00 |
| BAM7 | --- | --- | --- | --- |
| Baohuoside I | 8,49E+00 | 5,54 E+00 | 7,46E+00 to 9,70E+00 | 4,43E+00 to 6,93E+00 |
| Betulin | 9,43E+00 | 1,79 E+00 | 6,10E+00 to 1,73E+01 | 1,32E+00 to 2,45E+00 |
| Betulinic acid | 5,61E+00 | 2,21 E+00 | 3,97E+00 to 8,58E+00 | 1,47E+00 to 3,44E+00 |
| BH3I-1 | --- | --- | --- | --- |
| Bioymifi | 7,91E+00 | 5,00 E+00 | 5,80E+00 to 1,15E+01 | 3,62E+00 to 7,22E+00 |
| Birinapant | --- | --- | --- | --- |
| Bisdemethoxycurcumin | 1,16E+01 | 1,61E+01 | 9,64E+00 to 1,44E+01 | 1,42E+01 to 1,87E+01 |
| BV6 | 1,10E+01 | 6,09 E+00 | 8,73E+00 to 1,41E+01 | 3,90E+00 to 1,09E+01 |
| C87 | 2,96E+01 | 3,11E+01 | 2,28E+01 to 4,26E+01 | 1,95E+01 to 6,48E+01 |
| CBL0137 | 4,00E+00 | 3,26E+00 | 2,82E+00 to 2,61E+01 | 1,86E+00 to 6,72E+00 |
| CID2011756 | 9,09E+01 | 4,43E+01 | 5,06E+01 to 2,20E+02 | 2,12E+01 to 4,88E+02 |
| CID755673 | 1,24E+02 | 7,48E+01 | 6,20E+01 to 3,73E+02 | 3,60E+01 to 2,73E+02 |
| Citric acid | --- | --- | --- | --- |
| Columbianadin | --- | --- | --- | --- |
| COTI-2 | 9,92E-01 | --- | 3,14E-01 to 2,20E+00 | --- |
| CRT0066101 | 2,32E+00 | 8,72E-01 | 1,90E+00 to 2,85E+00 | 5,93E-01 to 1,24E+00 |
| Demethoxycurcumin | 1,84E+01 | 3,11E+01 | 1,53E+01 to 2,42E+01 | 1,79E+01 to 2,42E+02 |
| Elesclomol | 9,93E+01 | 3,75E+01 | 4,76E+01 to 3,95E+02 | 1,61E+01 to 2,04E+02 |
| Embelin | 3,27E+01 | 2,71E+01 | 2,40E+01 to 5,42E+01 | 2,31E+01 to 3,38E+01 |
| Epibrassinolide | --- | --- | --- | --- |
| Erastin | 4,21E+01 | 2,54E+01 | 2,98E+01 to 6,69E+01 | 1,08E+01 to 1,65E+02 |
| Ferrostatin-1 | --- | --- | --- | --- |
| FIN56 | --- | --- | --- | --- |
| Fisetin | --- | --- | --- | --- |
| FX1 | --- | --- | --- | --- |
| Gambogic Acid | 6,59E-01 | 6,25E-01 | 6,19E-01 to 7,01E-01 | 5,30E-01 to 7,30E-01 |
| GDC-0152 | --- | --- | --- | --- |
| Ginsenoside Rc | --- | --- | --- | --- |
| Ginsenoside Rh1 | --- | --- | --- | --- |
| Ginsenoside Rh2 | 2,44E+01 | 9,71E+01 | -∞ to 2,91E+01 | 2,62E+01 to 3,31E+03 |
| Glycochenodeoxycholic acid | --- | --- | --- | --- |
| Gossypol (acetic acid) | 1,21E+01 | 1,40E+01 | 1,04E+01 to 1,14E+02 | 9,56E+00 to 2,61E+01 |
| GSK’583 | 1,10E+02 | 1,27E+03 | 4,87E+01 to 5,01E+02 | 4,71E+01 to +∞ |
| GSK'481 | --- | --- | --- | --- |
| GSK'872 | 9,34E+01 | 2,72E+02 | 5,70E+01 to 1,90E+02 | 7,65E+01 to 7,44E+04 |
| HA14-1 | --- | --- | --- | --- |
| Homoplantaginin | --- | --- | --- | --- |
| Hypaconitine | --- | --- | --- | --- |
| Iberin | 3,44E+00 | 3,44E+00 | 3,04E+00 to 3,91E+00 | 3,17E+00 to 3,73E+00 |
| Inauhzin | 1,51E+01 | 5,29E+01 | 1,20E+01 to 1,99E+01 | 2,79E+01 to 1,47E+02 |
| Isoalantolactone | 8,69E+00 | 9,62E+00 | 7,56E+00 to 1,00E+01 | 7,81E+00 to 1,23E+01 |
| kb NB 142-70 | 1,21E+01 | 1,32E+01 | 9,02E+00 to 1,78E+01 | 7,46E+00 to 4,52E+00 |
| Kevetrin (hydrochloride) | --- | --- | --- | ---  --- |
| KJ Pyr 9 | 1,48E+02 | 1,68E+01 | 5,07E+01 to 1,34E+03 | 1,54E+01 to 1,84E+01 |
| LCL161 | 1,92E+02 | 1,32E+01 | 8,47E+01 to 7,30E+02 | 7,38E+00 to 3,70E+01 |
| Lenalidomide | --- | --- | --- | --- |
| Liproxstatin-1 | 1,41E+01 | 1,12E+01 | 1,21E+01 to 1,66E+01 | 8,28E+00 to 1,59E+01 |
| Marinopyrrole A | 2,49E+02 | 1,87E+02 | 5,36E+01 to 3,33E+04 | 3,38E+01 to 8,94E+04 |
| Mesaconitine | --- | --- | --- | --- |
| Methylprotodioscin | 8,89E+00 | 4,41E+00 | 7,87E+00 to 1,01E+01 | 2,90E+00 to 6,86E+00 |
| Methylthiouracil | --- | --- | --- | --- |
| MI-773 | 7,74E+00 | 7,48E+00 | 6,41E+00 to 9,54E+00 | 5,13E+00 to 1,22E+01 |
| Mulberroside A | --- | --- | --- | --- |
| Myricetin | --- | --- | --- | --- |
| **Navitoclax** | 5,69E+00 | 8,31E-01 | 4,94E+00 to 6,51E+00 | 7,06E-01 to 9,75E-01 |
| Necrostatin-1 | --- | --- | --- | --- |
| Neochlorogenic acid | --- | --- | --- | --- |
| NSC319726 | --- | --- | --- | --- |
| NSC348884 | 2,93E+00 | 2,45E+00 | 2,53E+00 to 3,33E+00 | 1,87E+00 to 3,16E+00 |
| NSC59984 | 1,63E+01 | 1,06E+01 | 1,45E+01 to 1,19E+02 | 7,51E+00 to 1,72E+01 |
| Nutlin (3) | 1,37E+01 | 1,01E+01 | 1,16E+01 to 1,67E+01 | 5,83E+00 to 2,44E+01 |
| Nutlin (3a) | 1,81E+01 | 1,25E+01 | 1,56E+01 to 1,22E+02 | 6,11E+00 to 6,53E+02 |
| Nutlin (3b) | 1,11E+01 | 6,70E+00 | 9,45E+00 to 1,33E+01 | 3,63E+00 to 1,77E+01 |
| Obatoclax | 3,96E+00 | 1,71E-02 | 2,57E+00 to 6,82E+00 | 1,56E-06 to 1,25E-01 |
| PAC-1 | 1,85E+01 | 9,72E+00 | 1,49E+01 to 2,45E+01 | 6,53E+00 to 1,72E+01 |
| Pifithrin-α (hydrobromide) | --- | --- | --- | --- |
| Pifithrin-β (hydrobromide) | --- | --- | --- | --- |
| Pifithrin-μ | --- | --- | --- | --- |
| Polydatin | --- | --- | --- | --- |
| Pomalidomide | --- | --- | --- | --- |
| PRIMA-1 | --- | --- | --- | --- |
| Puromycin aminonucleoside | --- | --- | --- | --- |
| QNZ | 2,52E-07 | 1,42E+00 | -∞ to 4,39E-01 | 2,05E-02 to 1,56E+01 |
| R-7050 | 4,20E+00 | 2,33E+00 | 3,47E+00 to 5,15E+00 | 1,80E+00 to 3,03E+00 |
| Raltitrexed | --- | --- | --- | --- |
| RG7112 | --- | --- | --- | --- |
| RG7388 | 2,62E+00 | 2,11E+00 | 2,29E+00 to 2,99E+00 | 1,59E+00 to 2,75E+00 |
| RIPA-56 | --- | --- | --- | --- |
| RITA | 6,61E+00 | 5,00E-02 | 1,82E+00 to 3,12E+03 | -∞ to 9,85E-01 |
| Roquinimex | --- | --- | --- | --- |
| Sanguinarine | 1,32E+00 | 1,25E+00 | 1,22E+00 to 1,42E+00 | 1,04E+00 to 1,50E+00 |
| SAR405838 | 5,28E+00 | 4,20E+00 | 4,03E+00 to 7,10E+00 | 2,61E+00 to 7,36E+00 |
| Serdemetan | 6,98E+00 | 4,01E+00 | 6,13E+00 to 7,95E+00 | 3,00E+00 to 5,38E+00 |
| Shikonin | 4,19E+00 | 6,18E+00 | 3,49E+00 to 5,07E+00 | 5,28E+00 to 5,72E+01 |
| SJ-172550 | --- | --- | --- | --- |
| Taurochenodeoxycholic acid | --- | --- | --- | --- |
| Tauroursodeoxycholate (sodium) | --- | --- | --- | --- |
| TC-DAPK 6 | --- | --- | --- | --- |
| Tenovin-1 | 5,12E+01 | 1,87E+01 | 1,90E+01 to 4,11E+02 | 6,45E+00 to 4,01E+02 |
| TIC10 | 3,58E+01 | 3,20E+01 | 2,12E+01 to 8,08E+01 | 1,35E+01 to 2,12E+02 |
| Trifluorothymidine | --- | --- | --- | --- |
| Ubiquitin Isopeptidase Inhibitor I | 2,94E+00 | 2,41E+00 | 2,09E+00 to 4,21E+00 | 1,50E+00 to 1,40E+01 |
| UMI-77 | 8,98E+00 | 6,73E+00 | 7,82E+00 to 1,04E+01 | 5,13E+00 to 8,24E+00 |
| VX-765 | --- | --- | --- | --- |
| WEHI-345 | 1,47E+01 | 1,31E+01 | 1,22E+01 to 1,82E+01 | 8,80E+00 to 2,31E+01 |
| YH239-EE | 8,16E+00 | 1,01E+01 | 7,29E+00 to 9,15E+00 | 7,63E+00 to 1,44E+01 |
| YM-155 | 9,33E-03 | 8,81E-06 | 2,89E-03 to 2,03E-02 | -∞ to 5,16E-03 |
